# Defining the impact of platelet-to-lymphocyte ratio on patient survival with gastric neuroendocrine neoplasm: a retrospective cohort analysis

**DOI:** 10.1186/s12957-022-02822-9

**Published:** 2022-11-09

**Authors:** Wenquan Liang, Xinxin Xu, Yuhua Liu, Jianxin Cui, Yunhe Gao, Chuang Wang, Ziwei Zhuang, Kecheng Zhang, Hongqing Xi, Aizhen Cai, Bo Wei, Lin Chen

**Affiliations:** 1 Department of General Surgery & Institute of General Surgery, The First Medical Center of Chinese PLA General Hospital, Beijing, 100853 China; 2 Medical School of Chinese PLA, Beijing, 100853 China; 3 Institution of Hospital Management, Department of Medical Innovation and Research, Chinese PLA General Hospital, Beijing, 100853 China

**Keywords:** Gastric neuroendocrine neoplasm, Platelet-to-lymphocyte ratio, Body mass index, Biomarker, Prognosis

## Abstract

**Background:**

Gastric neuroendocrine neoplasm (g-NEN) is a rare but heterogeneous neoplasm, with an increasing incidence yearly. Conventional prognostic markers of g-NEN remain limited which could only be detected after surgery. There is an urgent need to explore new prognostic markers for g-NEN patients. This study aimed to investigate the prognostic value of platelet-to-lymphocyte, ratio (PLR) and the association between PLR and body mass index (BMI) in patients with gastric neuroendocrine neoplasms (g-NEN).

**Methods:**

A retrospective cohort of patients with g-NEN from January 2001 through June 2016 was examined. The prognostic significance of PLR was determined by multiple regression analysis in different models. Stratified analysis was performed to examine the prognostic value of PLR at different BMI levels.

**Results:**

In total, 238 patients were enrolled. Those with higher PLRs tended to undergo open surgery, had larger tumor sizes, were diagnosed more frequently with neuroendocrine carcinoma, and had higher tumor grades. PLR was significantly associated with the survival of patients with g-NEN. With PLR increased per standard deviation, the all-cause mortality risk of patients with g-NEN increased by 67%, 63%, and 54% in the crude (HR = 1.67, 95% CI 1.32–2.12, *P* < 0.001), minimally adjusted (HR = 1.63, 95% CI 1.28–2.08, *P* < 0.001), and fully adjusted (HR = 1.54, 95% CI 1.202–1.98, *P* = 0.001) models, respectively. Patients with higher PLR (quartile 4, ≥ 187) had a 1.8-fold increase in all-cause mortality risk compared with those with lower PLR (quartile 1–3, < 187). Furthermore, there was a significant interaction effect between BMI subgroups and PLR in predicting the survival of patients with g-NEN (PLR regarded as a continuous variable: all *P* for interaction < 0.05 in the crude, minimally adjusted, and fully adjusted models; PLR regarded as a categorical variable: *P* for interaction < 0.05 in the fully adjusted model). Patients with g-NEN with the characteristics of higher PLR (quartile 4, ≥ 187) and non-obesity (BMI < 25 kg/m^2^) had worse survival than others (*P* < 0.05).

**Conclusion:**

The inflammation marker PLR has an independent prognostic value for patients with g-NENs, and high PLR combined with non-obesity increases the mortality risk of these patients.

## Background

Gastric neuroendocrine neoplasm (g-NEN) is a heterogeneous neoplasm with certain characteristic biological features. It is often indolent and generally slow-growing, but can also be aggressive and metastasize at an early stage [1]. G-NEN is a rare tumor, however, its incidence has been rising in recent years. An analysis of the Surveillance, Epidemiology, and End Result dataset from the USA suggested that the rate of g-NEN increased 15-fold from 1973 to 2012 [2]. The relevant research of g-NEN remains ambiguous and has gained the attention of clinical investigators [3]. Several clinicopathological factors, such as tumor size, invasion depth, and lymphatic and distant metastasis, have been demonstrated as critical prognostic factors in patients with g-NEN [4, 5]. However, the predictive value of these factors is limited, because they can only be evaluated after surgery. Therefore, better predictors that can be routinely evaluated before surgery at a low cost are required.

There is a growing consensus that the systemic inflammatory response from hematological components can be used to develop inflammation-based prognostic markers that are capable of predicting survival in several malignancies [6–8]. Among these widely studied inflammatory markers, the neutrophil-to-lymphocyte ratio (NLR) and platelet-to-lymphocyte ratio (PLR) have received increasing attention and have been demonstrated as independent prognostic markers for different cancers [8–12]. Systemic inflammation markers (including NLR and PLR) have been demonstrated to be associated with prognosis in gastroenteropancreatic neuroendocrine neoplasms (GEP-NENs) [13, 14]. For patients with g-NEN, two recent studies have shown that elevated NLR was associated with poor recurrence-free survival and overall survival (OS) [15, 16]. However, there is a lack of evidence regarding the prognostic value of PLR in g-NEN.

Several hypotheses have been proposed to explain the apparent “obesity paradox” in tumors, for example kidney and colon cancer, which suggests that obese patients with cancer show longer survival than those with normal weight [17, 18]. Moreover, previous evidence also revealed that decreased body mass index (BMI) combined with an elevated inflammatory marker (NLR) nearly doubled the mortality risk in nonmetastatic colorectal cancer [19]. A recent study reinforced obesity as an independent risk factor for GEP-NENs and suggested the role of metformin as a protective factor in diabetes [20]. To the best of our knowledge, the relationship between PLR and BMI in patients with g-NEN has not yet been reported.

Therefore, the objective of this study was to assess the independent prognostic value of PLR as a survival indicator in patients with g-NEN. Additionally, we plan to perform subgroup analyses to further examine the interaction effect of BMI and PLR in predicting patient survival.

## Methods

### Patient cohort

**Fig. 1:**
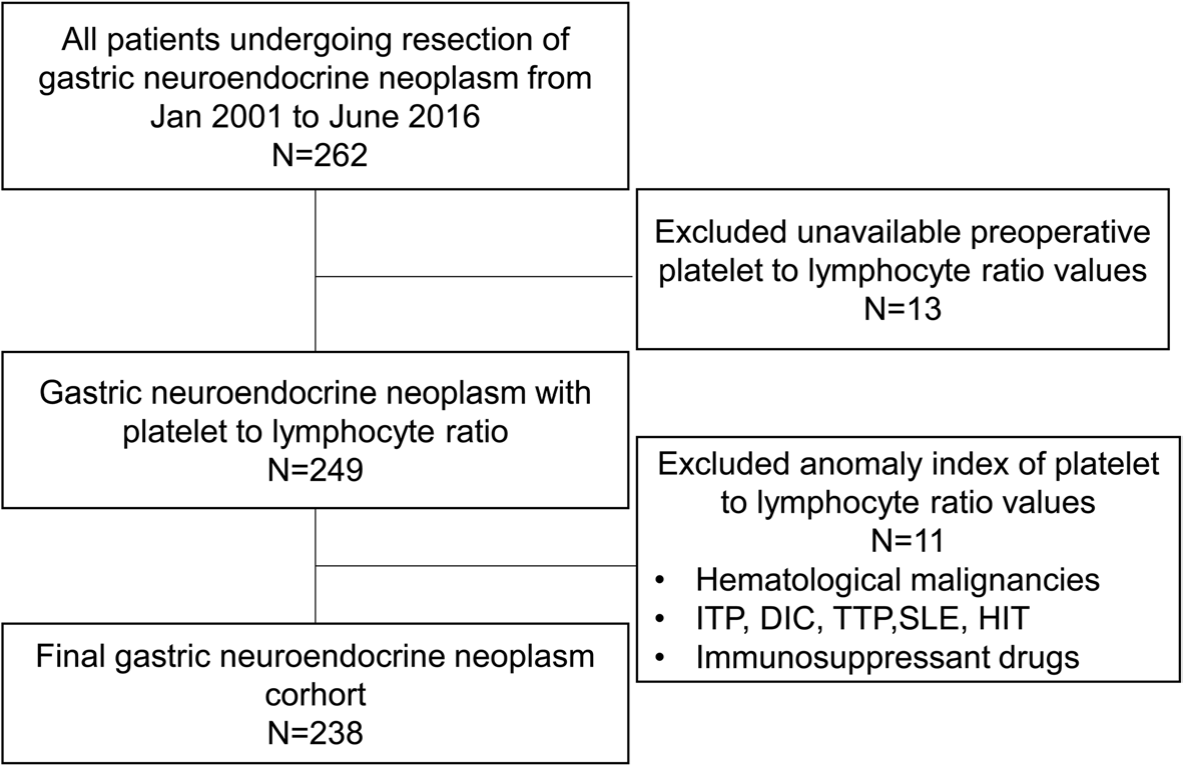
Patient enrolment flowchart. *Abbreviations*: ITP idiopathic thrombocytopenic purpura; DIC disseminated intravascular coagulation; TTP thrombotic thrombocytopenic purpura; SLE systemic lupus erythematosus; HIT heparin-induced thrombocytopenia

We performed a retrospective analysis of a stomach neoplasm database built by Chinese National Engineering Laboratory (Beijing, China) in the Chinese PLA General Hospital. A total of 262 consecutive patients who underwent lesionnectomy of pathologically confirmed g-NEN in PLA General Hospital from January 2001 to June 2016 were eligible for inclusion. Clinical diagnosis and treatment were performed entirely in accordance with the National Comprehensive Cancer Network guidelines. The inclusion criteria were (1) g-NENs confirmed by pathology; (2) patients without distant metastases who underwent radical resection, which included open surgery or minimally invasive surgery (endoscopic submucosal dissection, endoscopic mucosal resection, laparoscopic surgery, and robotic surgery); and (3) patients with complete follow-up data. The exclusion criteria were (1) unavailable preoperative PLR values (within 30 days of operation) and (2) treatment or disease that resulted in abnormal PLR values, including hematological malignancies, idiopathic thrombocytopenic purpura, disseminated intravascular coagulation, thrombotic thrombocytopenic purpura, systemic lupus erythematosus, heparin-induced thrombocytopenia, and use of immunosuppressants. The flow chart of participant enrollment is shown in Fig. 1. Due to the retrospective nature of the cohort analysis, informed consent was deemed unnecessary. This study was conducted in accordance with the Declaration of Helsinki, and the study protocol was reviewed and approved by the Institutional Review Board of the Chinese PLA General Hospital.

### Variable definitions

We obtained blood platelet (P) and lymphocyte counts (L) at baseline and recorded these values as continuous variables, and PLR was defined as P/L. Baseline height and weight (within 30 days of surgery) were measured and recorded as continuous variables. BMI was defined as the ratio of weight divided by height squared. Patients were classified into dichotomous groups according to the World Health Organization classification and separated into low BMI (< 25 kg/m^2^) and high BMI (≥ 25 kg/m^2^) cohorts. The covariates used in this study were selected based on the following: (1) demographic data, (2) variables that could affect survival as reported by previous literature, and (3) our clinical experiences. Therefore, the following variables were collected in this study: (1) continuous variables: age, platelet and lymphocyte counts, BMI, and tumor size (obtained from pathology reports); (2) categorical variables: sex, smoking status, chromogranin A (CgA), synaptophysin (Syn), tumor type and grading, T stage, N stage, M stage, and tumor stage (obtained from pathology reports). Tumor sizes were defined as the greatest diameter of the tumor, and those with continuous or cumulative smoking for 6 months or more in a lifetime were defined as smokers. Anti-CgA antibody (ab15160) and anti-Syn antibody (ab32127) were obtained from Abcam Trading Company, and two pathologists independently assessed whether the immunohistochemistry results were positive. TNM staging and tumor grading were based on the previous consensus and gastroenteropancreatic neuroendocrine tumor guidelines [21, 22]. A common classification framework for neuroendocrine neoplasms followed the World Health Organization (WHO) expert consensus proposal [23] and the Chinese consensus on the pathological diagnosis of gastrointestinal and pancreatic neuroendocrine neoplasms (2020) [24]. In short, neuroendocrine tumors were classified as neuroendocrine tumor (NET), neuroendocrine carcinoma (NEC), and mixed neuroendocrine/non-neuroendocrine neoplasm (MiNEN). Tumor grading was based on the Ki-67 index and mitotic count per 10 high-power fields.

### Follow-up procedure

The primary endpoint was OS, measured from the date of the surgery until the time of last follow-up or date of all-cause death. Follow-up was performed through outpatient visits, telephone surveys, and monitoring central death registries and publicly available obituaries. The follow-up interval was 6 months, and the items were regarded as censored values at 60 months or when information on survival time was incomplete. Follow-up data were managed by the second author and stored in the hospital’s electronic medical record system.

### Statistical analysis

Data are presented as the mean and standard deviation (SD) for continuous variables and frequency for categorical variables. The PLR quartiles were used as cutoff values for the analyses of categorical variables. The chi-square test (categorical variables), one-way ANOVA test (normal distribution), or Mann-Whitney *U* test (skewed distribution) was performed to evaluate differences among different PLR groups. The Kaplan-Meier log-rank test and Cox proportional hazards regression were employed to detect the relationship between variables and OS. Three models were constructed to identify the independent associations between PLR, BMI, and OS: crude model, no covariates were adjusted; model I, only sociodemographic data were adjusted; model II, covariates in model I and other elected covariates were adjusted. Variables were included in the fully adjusted model if the matched odds ratio changed by at least 10% as a result of adding those covariates, as described in previous studies [25]. Subgroup analyses were performed using stratified Cox proportional hazard models. Continuous variables were converted to categorical variables according to the clinical cutoffs or means and subsequently performed an interaction test between PLR and the variables to determine their relationship with OS. Tests for the effect modification for these subgroup indicators were followed by the likelihood ratio test. All analyses were performed with statistical software packages R (http://www.R-project.org, The R Foundation) and EmpowerStats (http://www.empowerstats.com; X&Y Solutions, Inc., Boston, MA, USA). *P* values less than 0.05 (two-sided) were considered statistically significant.

## Results

### Demographic characteristics and the relationship between PLR and clinical features

After screening according to the inclusion and exclusion criteria, 238 participants were selected for the final data analysis. The complete baseline characteristics of these selected participants were presented according to PLR quartiles (Q): Q1: < 103; Q2: ≥ 103 to < 137; Q3: ≥ 137 to < 187; and Q4: ≥ 187. The median age at the time of operation was 60 years, and approximately 75.21% of the patients were men. In the pooled analysis, there were no significant differences in baseline characteristics between each quartile, with the exceptions of operative approach and tumor size, type, and grade. Patients with higher PLR tended to undergo open surgery and showed associations with larger tumor size, NEC type, and higher tumor grade. The details of other baseline features, including sex, age, BMI, smoking status, CgA and Syn staining, and TNM tumor stage are shown in Table 1.

**Table 1:** Demographic features and association between PLR and clinical features

Variables	Total (*n* = 238)	Q1 (*n* = 60)	Q2 (*n* = 60)	Q3 (*n* = 60)	Q4 (*n* = 58)	*P* value
	(38–349)	< 103	> = 103–< 137	>= 137–< 187	> = 187	
PLR	152.64 ± 65.11	82.13 ± 16.03	121.51 ± 10.35	164.25 ± 14.34	245.78 ± 42.60	< 0.001
Sex						
Male	179 (75.21%)	42 (70.00%)	45 (75.00%)	51 (85.00%)	41 (70.69%)	0.204
Female	59 (24.79%)	18 (30.00%)	15 (25.00%)	9 (15.00%)	17 (29.31%)	
Age (years)						
Continuous	60.26 ± 10.09	58.18 ± 9.77	59.17 ± 10.86	62.78 ± 9.55	60.95 ± 9.76	0.063
<= 60	119 (50.00%)	34 (56.67%)	33 (55.00%)	25 (41.67%)	27 (46.55%)	0.307
> 60	119 (50.00%)	26 (43.33%)	27 (45.00%)	35 (58.33%)	31 (53.45%)	
BMI (kg/m^2^)						
Continuous	24.05 ± 3.35	24.66 ± 3.40	24.54 ± 3.14	23.64 ± 2.97	23.33 ± 3.76	0.076
< 25	139 (58.40%)	31 (51.67%)	29 (48.33%)	39 (65.00%)	40 (68.97%)	0.061
>= 25	99 (41.60%)	29 (48.33%)	31 (51.67%)	21 (35.00%)	18 (31.03%)	
Smoking status						
No	147 (61.77%)	43 (71.67%)	33 (55.00%)	33 (55.00%)	38 (65.52%)	0.16
Yes	91 (38.23%)	17 (28.33%)	27 (45.00%)	27 (45.00%)	20 (34.48%)	
Operative approach						
MIS	83 (34.87%)	28 (46.67%)	27 (45.00%)	13 (21.67%)	15 (25.86%)	0.004
Open surgery	155 (65.13%)	32 (53.33%)	33 (55.00%)	47 (78.33%)	43 (74.14%)	
Tumor size (cm)						
Continuous	4.24 ± 2.79	2.94 ± 1.91	3.90 ± 2.39	4.73 ± 2.95	5.45 ± 3.18	< 0.001
<= 4.0	112 (47.06%)	44 (73.33%)	28 (46.67%)	26 (43.33%)	14 (24.14%)	< 0.001
> 4.0	126 (52.94%)	16 (26.67%)	32 (53.33%)	34 (56.67%)	44 (75.86%)	
CgA						
Negative	127 (53.36%)	31 (51.67%)	33 (55.00%)	30 (50.00%)	33 (56.90%)	0.874
Positive	111 (46.64%)	29 (48.33%)	27 (45.00%)	30 (50.00%)	25 (43.10%)	
Syn						
Negative	28 (11.76%)	8 (13.33%)	5 (8.33%)	9 (15.00%)	6 (10.34%)	0.673
Positive	210 (88.24%)	52 (86.67%)	55 (91.67%)	51 (85.00%)	52 (89.66%)	
Tumor type						
NET	51 (21.43%)	18 (30.00%)	18 (30.00%)	7 (11.67%)	8 (13.79%)	0.015
NEC	137 (57.56%)	27 (45.00%)	31 (51.67%)	37 (61.67%)	42 (72.41%)	
MiNEN	50 (21.01%)	15 (25.00%)	11 (18.33%)	16 (26.67%)	8 (13.79%)	
Grade						
G1–G2	62 (26.05%)	23 (38.33%)	17 (28.33%)	12 (20.00%)	10 (17.24%)	0.04
G3	176 (73.95%)	37 (61.67%)	43 (71.67%)	48 (80.00%)	48 (82.76%)	
T stage						0.17
T1–T2	71 (29.83%)	23 (38.33%)	20 (33.33%)	16 (26.67%)	12 (20.69%)	
T3–T4	167 (70.17%)	37 (61.67%)	40 (66.67%)	44 (73.33%)	46 (79.31%)	
N stage						
N0	77 (32.35%)	21 (35.00%)	21 (35.00%)	21 (35.00%)	14 (24.14%)	0.5
N1–3	161 (67.65%)	39 (65.00%)	39 (65.00%)	39 (65.00%)	44 (75.86%)	
M stage						
M0	193 (81.09%)	53 (88.33%)	49 (81.67%)	49 (81.67%)	42 (72.41%)	0.177
M1	45 (18.91%)	7 (11.67%)	11 (18.33%)	11 (18.33%)	16 (27.59%)	
Tumor stage						
I–II	93 (39.08%)	27 (45.00%)	26 (43.33%)	23 (38.33%)	17 (29.31%)	0.298
III–IV	145 (60.92%)	33 (55.00%)	34 (56.67%)	37 (61.67%)	41 (70.69%)	
Chemotherapy						
No	182 (76.47%)	42 (70.00%)	44 (73.33%)	53 (88.33%)	43 (74.14%)	0.086
Yes	56 (23.53%)	18 (30.00%)	16 (26.67%)	7 (11.67%)	15 (25.86%)	

*Abbreviations*: *PLR* platelet to lymphocyte ratio, *Q* interquartile range, *BMI* body mass index, *MIS* minimally invasive surgery, *CgA* chromogranin, *Syn* synaptophysin, *NET* neuroendocrine tumor, *NEC* neuroendocrine carcinoma, *MiNEN* mixed neuroendocrine/non-neuroendocrine neoplasm

### Survival analysis

**Fig. 2:**
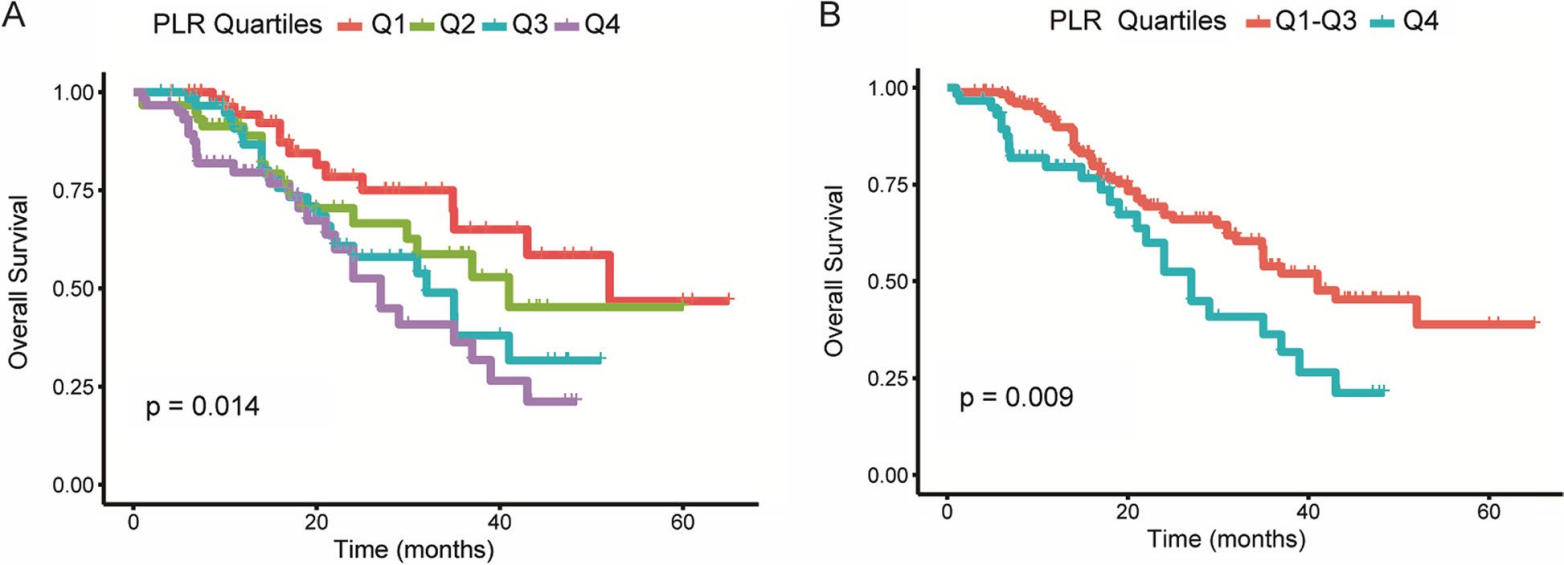
**A**, **B** Kaplan–Meier estimate of overall survival (OS) of patients with g-NEN according to PLR quartiles (Q1: < 103; Q2: >= 103 to < 137: Q3: >= 137 to < 187: and Q4: > = 187)

During follow-up, all-cause mortality was observed in 82 patients. Cox proportional hazards regression analyses were performed to assess the associations between PLR and BMI and OS in patients with g-NEN. Three models were constructed to analyze the independent effects of PLR on survival: the crude model, minimally adjusted model (model I), and fully adjusted model (model II). The tumor size and N stage were selected in the fully adjusted model because of a change of > 10% from the initial regression coefficient. This study showed that PLR was negatively associated with the survival of patients with g-NEN. The model-based effect size can be explained as a difference in the SD of PLR, which is associated with the mortality risk. As shown in Table 2, the PLR increased per SD, and the mortality risk of patients with g-NEN increased by 67%, 63%, and 54% in the crude (HR = 1.67, 95% CI 1.32–2.12, *P* < 0.001), minimally adjusted (HR = 1.63, 95% CI 1.28–2.08, *P* <0.001), and fully adjusted models (HR = 1.54, 95% CI 1.20–1.98, *P* = 0.001), respectively. Patients with the highest PLR (Q4) had a 2.55-fold increased risk of all-cause mortality compared with those in the lowest PLR group (Q1) in the fully adjusted model. However, the medium PLR groups (Q2 and Q3) were not significantly different from the lowest PLR group (Q1) in the fully adjusted model (all *P* values > 0.05). Based on this result, the highest PLR group (Q4) and the remaining three quartiles of PLR (Q1–Q3) were further analyzed, and the highest PLR group had a higher mortality risk in the crude, minimally adjusted, and fully adjusted models than the other three groups (all *P* values < 0.05). The Kaplan–Meier curves illustrate the cumulative mortality risks presented by the PLR quartiles (Fig. 2A, B). In contrast, BMI was not found to be associated with the survival of patients with g-NEN as a continuous or categorical variable (Table 2).

**Table 2:** Analysis of PLR in relation to overall survival in patients with g-NEN

Exposure	Crude model		Model I		Model II	
	HR (95% CI)	*P* value	HR (95% CI)	*P* value	HR (95% CI)	*P* value
PLR (per SD)	1.67 (1.32, 2.12)	< 0.001	1.63 (1.28, 2.08)	< 0.001	1.54 (1.20, 1.98)	0.001
PLR (quantile)						
Q1 (< 103)	Ref		Ref		Ref	
Q2 (>= 103–< 137)	1.58 (0.78, 3.18)	0.202	1.65 (0.82, 3.34)	0.162	1.59 (0.77, 3.30)	0.208
Q3 (>= 137–< 187)	2.03 (1.04, 3.96)	0.037	1.93 (0.97, 3.83)	0.060	1.63 (0.81, 3.30)	0.173
Q4 (>= 187)	2.77 (1.44, 5.34)	0.002	2.62 (1.35, 5.07)	0.004	2.55 (1.28, 5.09)	0.008
PLR (quantile)						
Q1–Q3 (< 187)	Ref		Ref		Ref	
Q4 (>= 187)	1.83 (1.15, 2.92)	0.011	1.73 (1.08, 2.76)	0.022	1.80 (1.10, 2.95)	0.020
BMI (continuous)	0.99 (0.94, 1.06)	0.959	1.01 (0.95, 1.07)	0.812	1.01 (0.95, 1.08)	0.782
BMI (categorical)						
< 25	Ref		Ref		Ref	
>= 25	0.88 (0.57, 1.36)	0.567	0.90 (0.58, 1.40)	0.641	0.98 (0.63, 1.53)	0.927

*Abbreviations*: *SD* standard deviation, *CI* indicates confidence interval, *HR* hazard ratio, *PLR* platelet to lymphocyte ratio, *IQR* interquartile range, *BMI* body mass index. The values in this table are expressed as the format of HR (95% CI). Crude model did not adjust covariant; *Model I* minimally adjusted for sex and age; *Model II* fully adjusted for sex, age, tumor size, and N stage

### Stratified subgroup analysis

A stratified analysis can be performed to examine the primary association of interest at different levels of a potential confounding factor. We employed the stratification variables to investigate the effect sizes of PLR on OS. The results showed that BMI had a significant stratified effect at different levels (Table 3). In non-overweight (BMI < 25 kg/m^2^) patients with g-NEN, the mortality risk increased by 2.02 times (95% CI 1.46–2.78, *P* < 0.001) per PLR SD increase. In contrast, preoperative PLR was not closely related to the mortality risk in the subgroup of patients with overweight (BMI ≥ 25 kg/m^2^). There was a significant interaction between PLR and BMI (*P* = 0.006). The other clinical features were well balanced, and no meaningful interactions with PLR were found (all *p* values > 0.05).

**Table 3:**
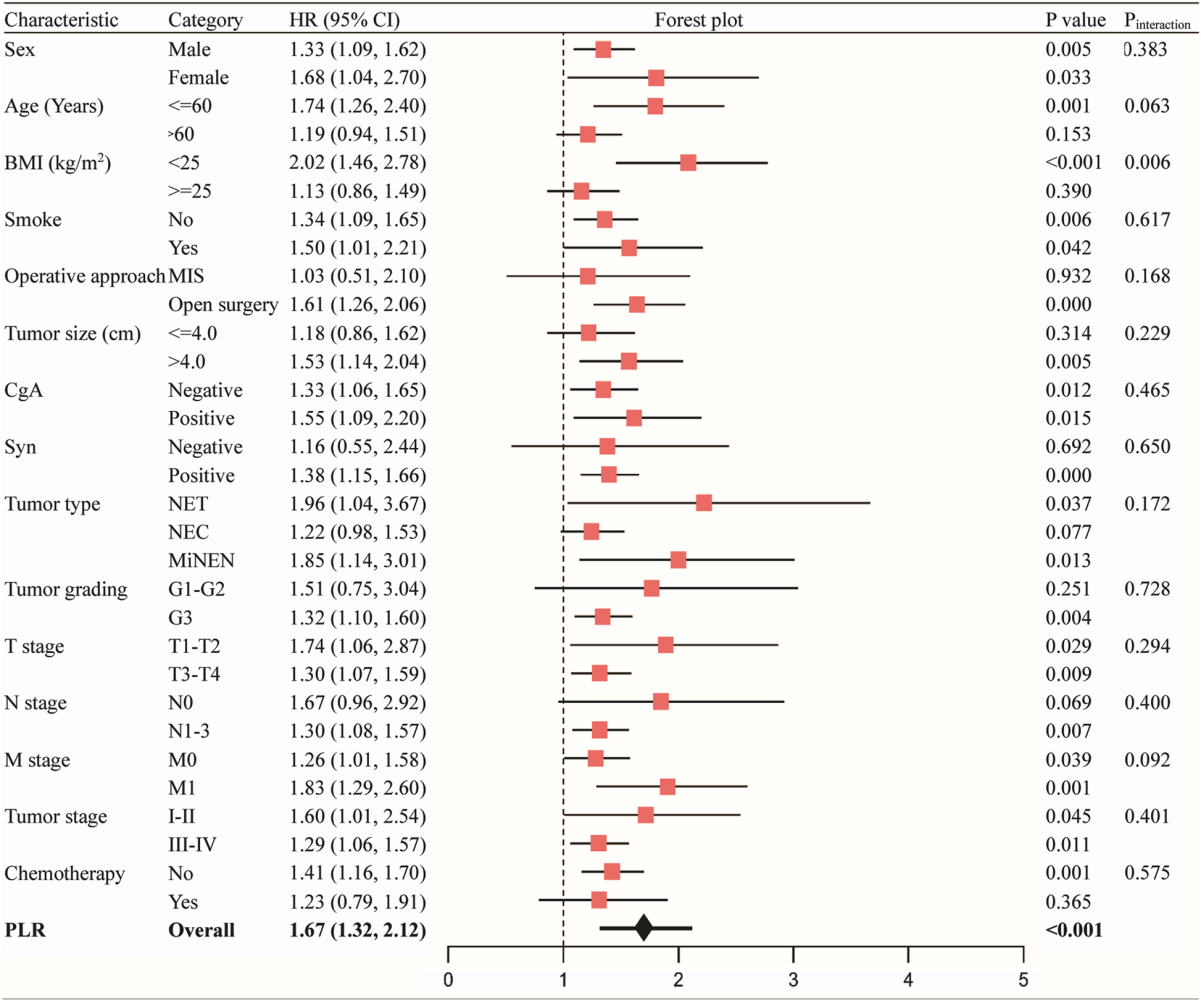
Stratified analysis of PLR (per SD) on OS of g-NEN

*Abbreviations*: *SD* standard deviation, *PLR* platelet to lymphocyte ratio, *BMI* body mass index, *MIS* minimally invasive technique, *CgA* chromogranin, *Syn* synaptophysin, *NET* neuroendocrine tumor, *NEC* neuroendocrine carcinoma, *MiNEN* mixed neuroendocrine/non-neuroendocrine neoplasmmixed adenoendocrine carcinoma

### PLR and BMI interaction analysis

**Fig. 3:**
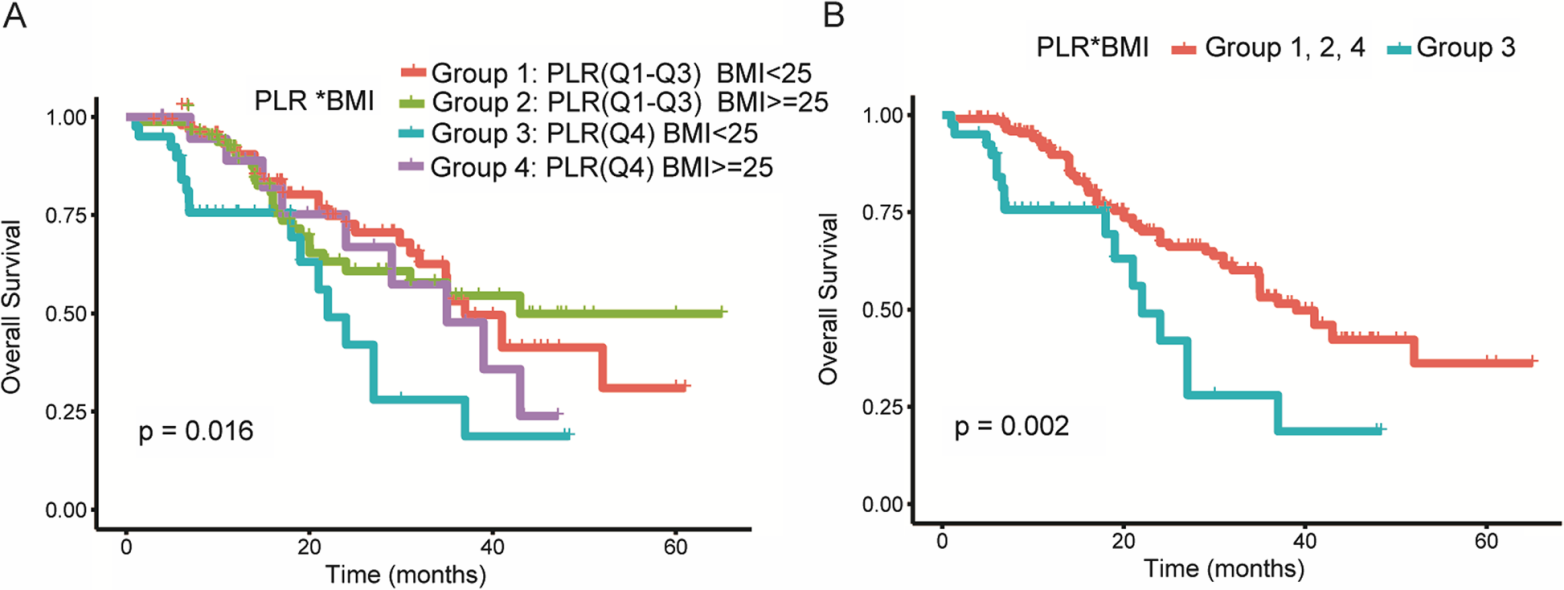
**A**, **B** Kaplan–Meier estimate of overall survival of patient with g-NEN according to PLR and BMI (group 1, PLR Q1–Q3 and BMI < 25 kg/m^2^; group 2, Q1–Q3 and BMI ≥ 25 kg/m^2^; group 3, PLR Q4 and BMI < 25 kg/m^2^; and group 4, PLR Q4 and BMI ≥ 25 kg/m^2^)

Table 4 shows the impact of BMI on the predictive efficacy of PLR for the OS of patients with g-NEN. For those with BMI < 25 kg/m^2^, PLR was negatively associated with OS, and the mortality risk increased significantly. In contrast, the mortality risk increased modestly with PLR in the high BMI subgroup (≥ 25 kg/m^2^), and this increase was not significant. Tests of the interactions between BMI subgroups and continuous PLR on OS were statistically significant (all *P* values < 0.05) in the crude, minimally, and fully adjusted models. We performed similar analyses to investigate the impact of PLR categories (Q4 vs Q1–3) on the OS of patients with g-NEN, which resulted in findings that were similar to those in the fully adjusted model (*P* = 0.047). However, the interactions between BMI subgroups and PLR categories were not significant in the crude (*P* = 0.179) and minimally (*P* = 0.159) adjusted models (Table 4), which indicated that the interactions between PLR and BMI were more apparent when related variables were adjusted. The PLR groups were analyzed according to BMI (< 25 or ≥ 25 kg/m^2^) in four cohorts as follows: group 1, PLR Q1–Q3 and BMI < 25 kg/m^2^; group 2, Q1–Q3 and BMI ≥ 25 kg/m^2^; group 3, PLR Q4 and BMI < 25 kg/m^2^; and group 4, PLR Q4 and BMI ≥ 25 kg/m^2^. Figure 3A shows the cumulative probabilities of deaths according to these four groups (*P* = 0.016). In group 3, patients with g-NEN with higher PLR and lower BMI presented more dramatic mortality risks than those in the remaining groups. We further compared the cumulative probability of death between group 3 and the remaining three groups (groups 1, 2, and 4) (Fig. 3B), and the cumulative difference in survival rate was more significant (*P* = 0.002).

**Table 4:** Stratified analyses of the interaction between PLR and BMI in relation to overall survival in patients with g-NEN

	Crude model		*P*_interaction_	Model I		*P*_interaction_	Model II		*P*_interaction_
	HR (95% CI)	*P* value		HR (95% CI)	*P* value		HR (95% CI)	*P* value	
PLR (per SD)			0.006			0.008			0.007
BMI < 25	2.02 (1.46, 2.78)	< 0.001		1.96 (1.42, 2.72)	< 0.001		1.95 (1.38, 2.76)	< 0.001	
BMI >= 25	1.13 (0.86, 1.49)	0.390		1.12 (0.84, 1.48)	0.448		1.04 (0.75, 1.44)	0.815	
PLR (Q4 vs Q1–3)			0.179			0.159			0.047
BMI < 25	2.45 (1.33, 4.48)	0.004		2.38 (1.29, 4.39)	0.005		2.22 (1.14, 4.30)	0.019	
BMI >= 25	1.25 (0.58, 2.66)	0.569		1.17 (0.55, 2.51)	0.682		1.44 (0.64, 3.26)	0.378	

*Abbreviations*: *SD* standard deviation, *CI* indicates confidence interval, *HR* hazard ratio, *PLR* platelet to lymphocyte ratio, *IQR* interquartile range, *BMI* body mass index. The values in this table are expressed as the format of HR (95% CI) *P* value

Crude model did not adjust covariant

Model I minimally adjusted for sex and age

Model II fully adjusted for sex, age, tumor size, and N stage

## Discussion

The findings of the present study indicated that preoperative PLR was an independent predictor of OS in patients with g-NEN undergoing surgical resection. The preoperative PLR increased per SD, and the mortality risk of patients with g-NEN tumors increased by 54–67%; significant differences in survaddival were observed among dichotomized PLR subgroups. In addition, we found variability in the predictive efficacy of preoperative PLR for OS across stratified BMI subgroups. A stronger association between preoperative PLR and survival was detected in non-overweight (BMI < 25 kg/m^2^) patients with g-NEN, and this association increased the mortality risk by 95% per PLR SD increase in the fully adjusted model. In contrast, preoperative PLR was not closely related to OS in the subgroup of overweight (BMI ≥ 25 kg/m^2^) patients with g-NEN. Our data indicated that preoperative PLR had remarkable prognostic value in non-overweight patients with g-NEN. These findings, if further confirmed in prospective studies, will help identify individuals at a high risk of mortality before surgery.

G-NEN is a rare digestive system tumor that accounts for 1% of gastric tumors. Its incidence has increased four-fold over the past 50 years, with the widespread use of gastroscopy and biopsy [26]. Conventional g-NEN prognostic markers are limited, and the tumors can only be detected after surgery [27, 28]. PLR and NLR have been regarded as inflammatory markers that reflect immune reactions and are related to poor OS in patients with some malignancies [7, 9, 11, 29]. In previous studies by Cao et al., NLR has been demonstrated to be a significant prognostic factor in patients with g-NEN [15, 16]. This study revealed that PLR was an independent prognostic factor associated with OS in patients with g-NEN in different models. In this study, the risk of mortality increased per PLR increase with SD, and the mortality risk of patients with g-NEN increased by 54% in the fully adjusted model. Consistent with our conclusions, Chen et al. also demonstrated the useful predictive ability of PLR in patients with oral squamous cell carcinoma and found that patients with higher PLR presented a 1.8-fold increased risk compared with those with lower PLR [30]. In a systematic review and meta-analysis that included 1904 patients with pancreatic cancer, the pooled analysis demonstrated that elevated PLR (> 150) was associated with decreased OS, particularly in Asian populations [31]. Moreover, Xu et al. conducted a meta-analysis comprising 4513 patients with gastric cancer and found that elevated PLR correlated with higher risks of lymph node metastasis, serosal invasion, and advanced stage [32].

This study did not aim to provide a mechanistic understanding of why PLR had prognostic value in patients with g-NEN. In general, platelets protect tumor cells from cytolysis and promote metastasis by glycoprotein bridging [33]. Increasing platelet levels can promote tumor growth due to increased levels of several angiopoietin factors and tumor cell extravasation [34]. Moreover, a high PLR is associated with lymphocytopenia, and a low lymphocyte count is linked to inadequate defense against cancer [35]. Further research should be conducted to investigate the effects of PLR on tumor immunity and its relevant molecular mechanisms.

This study revealed interesting associations between PLR and clinicopathological factors. The subgroup analysis helped us understand survival trends according to PLR in different populations. Our results confirmed that BMI at baseline had a strong stratified effect on the predictive ability of preoperative PLR, and a significant association between PLR and OS was found in non-overweight patients. However, no significant associations were found after adjusting for confounding factors in overweight patients. BMI, a common indicator of nutritional status, may strongly affect systemic immune function [36]. Previous studies have confirmed the association of increased BMI with higher risks of esophageal and gastric cardia adenocarcinoma in Western populations [37]. However, the study by Tran et al. found no association between BMI and gastric cardia adenocarcinoma in a Chinese population, which had a different BMI distribution [38]. Recently, a study investigated obesity as an early predictor of GEP-NET and revealed that higher values of obesity were significantly correlated with the worst clinical severity in NENs [39]. In the present study, BMI was not correlated with the survival of patients with g-NEN, either as a continuous or categorical variable.

To the best of our knowledge, this is the first study to examine the relationship between PLR and BMI in a survival analysis of patients with g-NEN and the only one to examine the stratified effects of the primary association between PLR and OS at different BMI levels. These findings should help future research on the establishment of diagnostic and predictive models for the survival of patients with g-NEN. Our study also had the following strengths. First, the studied continuous and categorical variables were evaluated for associations with OS. Second, this study was non-interventional in nature and was therefore susceptible to the limitation of potential confounding, although we used strict statistical adjustment to minimize residual confounders, but such an approach can reduce the contingency in the data analysis and enhance the robustness of the results. Finally, the effect modifier factor analysis resulted in better utilization of data and yielded stable conclusions in the different subgroups in this study.

This study had several limitations that require further discussion. First, this study was retrospective in nature and conducted in a single institution. Second, our research participants were Chinese patients with g-NENs; therefore, there is a certain limitation to the universality and extrapolation of our results. Third, the liver is the main site of g-NEN metastases as recently published [40]. The primary endpoint of this study was OS and the data on liver metastases was lacking. Finally, our investigation of the stratified effect of BMI lacked a validation cohort. Hence, these are only hypothesis-generating findings and require additional validation in a more extensive prospective study.

## Conclusion

In conclusion, the findings of our study suggest that preoperative PLR was an independent predictor of OS in patients with g-NEN undergoing surgical resection. There was an interaction between PLR and BMI in predicting survival, and PLR had remarkable prognostic value in non-overweight patients with g-NEN. PLR and BMI are routinely measured in clinical practice and thus, have potential value in predicting the survival of patients with g-NEN. This study provides a foundation for future research on inflammation markers and BMI as a prognostic parameter in clinical cancer research.

## Data Availability

The data that support the findings of this study are available from the corresponding author upon reasonable request.

## Abbreviations

PLR: Platelet to lymphocyte ratio
*Q*: Interquartile range
BMI: Body mass index
MIS: Minimally invasive surgery
CgA: Chromogranin
Syn: Synaptophysin
NET: Neuroendocrine tumor
NEC: Neuroendocrine carcinoma
MANEC: Mixed adenoendocrine carcinoma
SD: Standard deviation
CI: Indicates confidence interval
HR: Hazard ratio
IQR: Interquartile range
ITP: Idiopathic thrombocytopenic purpura
DIC: Disseminated intravascular coagulation
TTP: Thrombotic thrombocytopenic purpura
SLE: Systemic lupus erythematosus
HIT: Heparin-induced thrombocytopenia
